# Assessment of Depression and Anxiety Among Admitted People With Heart Disease Conditions: A Cross-Sectional Hospital-Based Study in a Bangladeshi Population During the COVID-19

**DOI:** 10.3389/fpsyt.2022.895224

**Published:** 2022-07-07

**Authors:** Mohammad Ashraful Amin, Mohsin Ahmed, Sabrina Nahin, Nadira Sultana Kakoly

**Affiliations:** ^1^Department of Public Health, North South University, Dhaka, Bangladesh; ^2^Public Health Professional Development Society (PPDS), Dhaka, Bangladesh; ^3^Department of Cardiology, National Institute of Cardiovascular Diseases (NICVD), Dhaka, Bangladesh; ^4^Department of Physiology, Green Life Medical College Hospital, Dhaka, Bangladesh

**Keywords:** anxiety, cardiovascular diseases, COVID-19, depression, Hospital Anxiety and Depression Scale (HADS), risk factors

## Abstract

**Objective:**

Depression and anxiety are widespread and chronic among patients with heart disease. We wanted to determine the proportion of heart patients with depression and anxiety levels as well as factors contributing toward depression and anxiety among hospitalized heart disease patients in Dhaka, Bangladesh during the COVID-19 era.

**Methods:**

The study comprised a total of 384 participants with a confirmed heart disease diagnosis. We conducted a cross-sectional study from 5th March to 27th June 2021. The hospital-based study admitted patients sequentially with a new or pre-existing heart disease diagnosis to one of Dhaka's two leading hospitals. The Hospital Anxiety and Depression Scale screened all individuals for depression and anxiety.

**Result:**

Most of the respondents (88.2%) were male and within the age categories of 51–60 years (32.81%). 96.6% of the patients were married, 30% had no income, 36.6% had only completed classes 1–5, and ~47% resided in rural areas. Approximately 36% of the study participants were former smokers, with 31% current smokers. Borderline abnormal and abnormal levels of anxiety and borderline abnormal and abnormal levels of depression were found in (23.9%, 49.4%) and (55.7%, 13.3%), respectively, of hospitalized patients. Age, residence, profession, monthly income, and chronic disease were significant predictors of anxiety, while only gender remained significantly associated with depression.

**Conclusion:**

Hospitalized Bangladeshi patients with heart disease had moderate levels of depression and anxiety. There is a need to develop a quick screening approach in hospitals dealing with hospitalized patients with heart disease to identify those needing extra evaluation and care.

## Introduction

Cardiovascular disease (CVD) is responsible for more deaths each year than any other cause, accounting for 32% of worldwide fatalities. Three-quarters of CVD deaths occur in low-and middle-income nations (1, 2). Non-communicable diseases (NCDs) account for an estimated 59% of total deaths in Bangladesh—around 886,000 annual deaths (1). Bangladesh is experiencing a colossal threat of NCDs, where 30% of all NCD mortality cases are accounted for by CVD (3).

Psychiatric morbidities, such as depression and anxiety, are prevalent in patients with heart disease (4). For several decades, clinicians recognized anxiety and depression in patients with heart problems, including patients with heart attack, heart failure, as well as systemic hypertension, particularly in those patients hospitalized in the coronary care unit (4–7). It is anticipated that by 2020, ischemic heart disease and depression will become the first and second contributors to health impairment and mortality globally (8). According to a research brief provided by the World Health Organization (WHO), the global prevalence of anxiety and depression surged by a massive 25% in the first year of the COVID-19 pandemic. Although the situation had improved by the end of 2021, far too many people continue to be unable to receive the care and assistance they require for both pre-existing and newly established mental health issues (9).

Despite anxiety is a natural and anticipated reaction to a heart attack or the dangers of living with a chronic condition, persistent or severe anxiety is not normal and has significant health implications for individuals (5, 10–12). Detection and treatment of the psychiatric condition (anxiety and depression) in patients affected with coronary artery disease (CAD) have been shown to enhance the CAD patients' survival rate and life expectancy (7). Potential risk adjustments, prescription medications, and recovery plans can be best adhered to by patients receiving care for their depression and anxiety (13).

Major depressive disorder (MDD), basically limited to depression, is a frequently diagnosed psychiatric condition with more than 300 million people globally affected and has been linked with an elevated risk of coronary heart disease (CHD) (14). In CVD patients, the prevalence of depression has been found to be around 15–30% (14), which is two to three percentage points higher than in the average community. However, healthcare systems have not been adapted adequately, with fewer than 15% of heart patients identified and treated for depression (15). Depression can lead to poor drug compliance, and the cardiovascular consequences of poor compliance have a bad prognosis. As a result, individuals with known CAD and psychiatric illnesses should be assessed (16) which can enhance the psychological health benefits of individuals with or at risk of cardiovascular disease.

In other countries, psychological problems are stigmatized (17). The incidence of anxiety and depression among heart patients in low-income countries is little known (18), including in Bangladesh. A countrywide survey found that Bangladesh has a high prevalence of mental health problems and inadequate mental health facilities (19). In Bangladesh, despite a rise in their incidence, mental illnesses remain undiagnosed, evaluated, or managed, and cardiovascular disease is still the primary cause of death. Unmet mental health requirements may be a significant roadblock to optimal heart disease patient management (20–23). Given the gaps in knowledge and significant health consequences, the current study aims to determine the proportion of heart patients with depression and anxiety levels as well as factors contributing toward depression and anxiety among hospitalized heart disease patients in Dhaka, Bangladesh.

## Materials and Methods

### Study Design and Population

A hospital-based cross-sectional study was conducted. Two central cardiology hospitals, one government and one private hospital in Dhaka, Bangladesh, were selected for this study.

**Figure 1:**
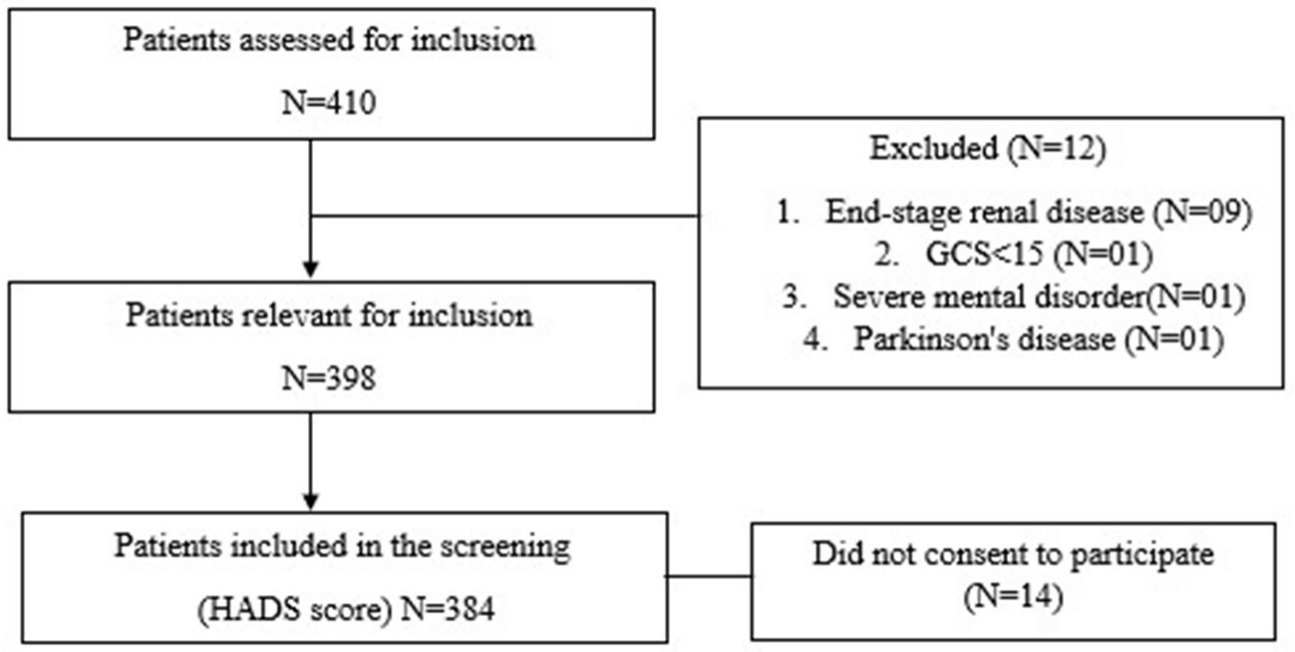
Flowchart of patients assessed for inclusion, excluded, and included patients, HADS, Hospital Anxiety and Depression Scale.

Patients were selected based on the pre-requirements of being aged between 18 and 80 years and having a confirmed or suspected heart disease diagnosis requiring hospitalization between March to June 2021. Our outcome variables included systemic hypertension, CAD, MI (Both ST elevation and non-ST elevation), angina, Heart failure, cardiac arrhythmia, valve pathology, as well as any other type of heart disease. Medical records from the hospital were used to validate heart disease diagnoses. Hospitalized heart disease patients routinely admitted with a history of pre-existing heart disease or newly diagnosed patients with heart disease admitted to the Cardiology Unit of the hospital were enrolled in the study. Those patients seen in an outdoor cardiac department in the hospital were excluded. We also excluded participants if they had a previously normal cardiac catheterization (CATH), an acute or previous stroke, end-stage renal disease (including dialysis patients), troubles with the nervous system (dementia, Alzheimer's disease, epilepsy, Parkinson's disease), mental retardation, a severe and frequently major psychological disorder, or any other serious clinically diagnosed mental health disorder. The study's objectives were communicated to admitted patients, and those who decided to participate gave their signed informed consent. A BMDC (Bangladesh Medical and Dental Council certified), the nationally recognized doctor, selected eligible patients during patient admission based on inclusion criteria. Later on, interviews were done based on a questionnaire and validated scale for this study. Patients were only enrolled once they had recovered from their acute crises and could consent (Figure 1). Hospital records and medical information were also used to collect data. Patients were interviewed while undergoing therapy (after CATH) or after treatment within 1 week of admission to the hospital.

Zigmond and Snaith (24) invented the Hospital Anxiety and Depression Scale (HADS), which is widely used worldwide. The Hospital Anxiety and Depression Scale (HADS) measured anxiety and depression. The HADS scale, a self-reported assessment instrument, was applied to detect anxiety and depressive symptoms among hospitalized admitted indoor cardiology patients using a Bengali version of the scale (25). This questionnaire has the HADS-A and HADS-D subscales, measuring anxiety and depressive symptoms. Seven items were used to measure depression, and seven items were used to measure anxiety. Each questionnaire topic was given a severity rating based on a four-point Likert scale ranging from 0 (not at all) to 4 (intense). Each sub-scale has the highest score of 21, and the full scale has a total score of 42, which might indicate emotional disorder; subscale scores range from 0 (no symptoms) to 21 (maximum of symptoms). A classification scheme can also be used to grade the evaluation: a score of 0–7 implies no clinical symptoms, 8–10 indicates moderate depression or anxiety, and a score of 11–21 shows diagnostic depression or anxiety (26). All major European languages and Arabic, Hebrew, Chinese, Japanese, and Urdu have HADS score translations; interpretations of other languages are also possible. The Bengali version of the HADS scale, a self-reported screening tool, was subsequently used in Bangladesh to assess anxiety and depression symptoms across various patients (27, 28). In study, The HADS translation from English to Bangla was evaluated by five independent reviewers fluent in both English and Bangla. On a scale of one to four, the comparability of the translation was assessed, with higher numbers indicating better comparability (25). We also conducted a pilot study with 20 hospital patients to determine the validity and reliability of the HADS score for the Bangla version. Three hundred eighty-four patients were identified as potential respondents (Figure 1).

This scale has been evaluated in various medical settings and has a high level of reliability (mean Cronbach's alpha = 0.83 for HADS-A and mean Cronbach's alpha = 0.82 for HADS-D) (29). HADS-D had a Cronbach's alpha of 0.71, whereas HADS-A had a Cronbach's alpha of 0.70 in this analysis.

## Ethics Statement

All participants signed a permission form and provided their informed consent. North-South University's Institutional Review Board/Ethical Review Committee also provided ethical approval for this project (2021-IRB-0502).

## Statistical Analysis

The proportion of heart patients with depression and anxiety levels and predictors of depression and anxiety among heart disease hospitalized patients were the key outcome measures. To summarize the patient's demographic characteristics, anxiety, and depression, descriptive statistics such as frequency, percentage and standard deviation were calculated. The Chi-square test assessed the relationship between anxiety and depression levels and socioeconomic and demographic variables. Fisher's exact test was used to assess the association for any contingency table less than five cell frequency.

The relationship between predictors and depression or anxiety was investigated using binary logistic regression. Multiple regression was used to adjust for those predictors significantly associated with the binary model. We reported odds ratios (ORs) and 95% confidence intervals (CIs) of indicators associated with mild and severe levels of depression or anxiety. All *p*-values <0.05 were considered as statistically significant. STATA (V16) was used to manage the data and conduct analyses. Internal consistency of the various scales was assessed using Cronbach alpha.

## Results

From March 2021 to June 2021, all patients who met the inclusion criteria were included in the study. We enrolled 384 heart disease patients from two hospitals, one govt Hospital—the National Institute of Cardiovascular Diseases (NICVD) and another private medical college & hospital. Among these two hospitals, 337 patients were enrolled from NICVD and 47 patients from private medical colleges & hospitals.

## Socio-Demographic Factors

Demographic characteristics are presented in Table 1. Among the 384 patients, 88.2% were males and within the age groups of 51–60 years (32.81%). Most participants were married (96.6%), with 27.4% in the business profession. 30% of patients had no income and 36.6% had only education of up to class five. Most of the study population lived in rural areas (46.61%) (Table 1).

**Table 1:** Socio-demographic and lifestyle characteristics of variables of the respondents, (*N* = 384).

**Variables**	**Total number of participants (*N =* 384)**	**Percentage (%)**
**Age**
Up to 40 years	63	16.41
41–50 years	106	27.60
51–60 years	126	32.81
61 to above years	89	23.18
**Gender**
Male	339	88.28
Female	45	11.72
**Religion**		
Muslim	350	91.15
Hindu	31	8.07
Others	03	0.78
**Residence**
Urban	159	41.41
Rural	179	46.61
Semi-rural	46	11.98
**Monthly income**
1- <10,000 taka	73	19.01
10,000–20,000 taka	93	24.22
20,001–50,000 taka	85	22.14
>50,000 taka	17	4.43
No income	116	30.21
**Marital status**
Married	371	96.61
Unmarried	12	3.13
Divorced/widow	01	0.26
**Occupation**
Unemployment	52	13.54
Government job	11	2.86
Private job	94	24.48
Businessman	105	27.34
Farmer	47	12.24
Retired	33	8.59
Housewife	42	10.94
**Education**
No formal education	44	11.46
Class 1–5	140	36.46
Class 6–10	125	32.55
Class 11–12	43	11.20
Graduation	26	6.77
Post-graduation	06	1.56
**Division**
Dhaka	239	62.24
Chittagong	74	19.27
Khulna	15	3.91
Sylhet	12	3.13
Rajshahi	10	2.60
Rangpur	15	3.91
Barishal	09	2.34
Mymensingh	10	2.60
**BMI**
Underweight <18.5	17	4.43
Normal 18.5–25	164	42.71
Overweight 25–30	108	28.13
Obese 30–40	95	24.74

## Behavior Factors

Almost 36% of those who were enrolled, were former smokers, with 31% current smokers. Alcohol usage was few, with 97% of subjects reporting no alcohol intake. Around 53% of patients were either overweight or obese (Table 1).

## Clinical Factors

The primary heart disease diagnoses among the patients were STEMI (46.6%), NSTEMI (17.2%), old MI (18.5%), and other diagnoses around (18%), including mitral or aortic valve stenosis, valve disease and heart block. Diagnosed patients had comorbidities such as chronic disease of systemic hypertension 17.71%, Diabetes mellitus 13.54%, and a combination of both 9.90% (Table 2).

**Table 2:** Clinical diagnosis and cardiac symptoms of the respondents, (*N* = 384).

**Clinical diagnosis**	**Total number of participants *N =* 384**	**Percentage (%)**
STEMI	179	46.61
NSTEMI	66	17.19
Old MI	71	18.49
RMI	15	3.91
Unstable angina	74	19.27
Stable angina	11	2.86
Systemic hypertension	99	25.78
Diabetes mellitus	84	21.88
ALVF	59	15.36
Complete heart block	15	3.91
H/O PCI	17	4.43
H/O CABG	07	1.82
Heart valve disease	26	6.77
Arrhythmia	17	4.43
ICM	18	4.69
**Symptoms**
Chest pain	309	80.47
Dyspnea	51	13.28
Cough	48	12.50
Palpitation	41	10.68
Edema	15	3.91
Orthopnea	12	3.13
Vomiting	16	4.17
Insomnia	24	6.25
No	46	11.98

### The Prevalence of Heart Disease Patients With Different Levels of Depressive and Anxiety

Even with mild symptoms, a HADS score of more than eight appears to be the best cut-off point for screening for anxiety and depression. Depressive symptoms were categorized as borderline abnormal and abnormal, and anxiety symptoms were categorized as borderline abnormal and abnormal anxiety symptoms. A total of 24% and 49% suffered from borderline abnormal and abnormal anxiety, respectively, and 56% and 14% had borderline abnormal and clinical depression, respectively (Table 3).

**Table 3:** Proportion of heart patients with depression and anxiety levels patients among hospitalized, (*N* = 384).

**Anxiety Level**	**Total number of participants (*N =* 384)**	**Percentage (%)**
Normal	102	26.56
Borderline abnormal	92	23.96
Abnormal	190	49.48
**Depression level**
Normal	119	30.99
Borderline abnormal	214	55.73
Abnormal	51	13.28

Males had borderline abnormal and abnormal anxiety in around 24% and 51% of cases, respectively, and females had borderline abnormal and abnormal anxiety in about 25% and 36% of cases. Male respondents revealed a higher abnormal degree of depression than female, with 5% in females and 15% in males. Similarly, graduate respondents and Government service holders had elevated levels of abnormal depression than other respondents. Interestingly homemakers also displayed a significant degree of borderline depression compared to other professions. In terms of income, persons earning 20,001–50,000 tk had the largest number (30%) of abnormal degree depression, while those who did not earn had the lowest number (5%) (Table 4).

**Table 4:** Association of the level of anxiety and depression with different variables.

**Variables**	**Category**	**Anxiety**				**Depression**			
		**Normal**	**Borderline abnormal**	**Abnormal**	***p***	**Normal**	**Borderline abnormal**	**Abnormal**	***p***
Gender	Female	18 (40.00)	11 (24.44)	16 (35.56)	0.065	8 (17.78)	35 (77.78)	2 (4.44)	**0.006**
	Male	84 (24.78)	81 (23.89)	174 (51.33)		111 (32.74)	179 (52.80)	49 (14.45)	
Age	Up to 40	17 (26.98)	16 (25.40)	30 (47.62)	0.207	26 (41.27)	27 (42.86)	10 (15.87)	0.000^*^
	41–50	31 (29.25)	17 (16.04)	58 (54.72)		40 (37.74)	41 (38.68)	25 (23.58)	
	51–60	27 (21.43)	33 (26.19)	66 (52.38)		34 (26.98)	82 (65.08)	10 (7.94)	
	61 to above	27 (30.34)	26 (29.21)	36 (40.45)		19 (21.35)	64 (71.91)	6 (6.74)	
Religions	Islam	92 (26.29)	83 (23.71)	175 (50.00)	0.817	109 (31.14)	196 (56.00)	45 (12.86)	0.670
	Hinduism	10 (32.26)	8 (25.81)	13 (41.94)		10 (32.26)	16 (51.61)	5 (16.13)	
	Buddhism	0 (0.00)	1 (50.00)	1 (50.00)		0 (0.00)	1 (50.00)	1 (50.00)	
	Christianity	0 (0.00)	0 (0.00)	1 (100.00)		0 (0.00)	1 (100.00)	0 (0.00)	
BMI (kg/m^2^)	Underweight	6 (35.29)	7 (41.18)	4 (23.53)	0.008^*^	5 (29.41)	12 (70.59)	0 (0.00)	0.145
	Normal	47 (28.66)	34 (20.73)	83 (50.61)		54 (32.93)	84 (51.22)	26 (15.85)	
	Overweight	18 (16.67)	24 (22.22)	66 (61.11)		25 (23.15)	69 (63.89)	14 (12.96)	
	Obese	31 (32.63)	27 (28.42)	37 (38.95)		35 (36.84)	49 (51.58)	11 (11.58)	
Residence	Rural	53 (29.61)	44 (24.58)	82 (45.81)	0.031^*^	59 (32.96)	100 (55.87)	20 (11.17)	0.226
	Semi-Urban	6 (13.04)	7 (15.22)	33 (71.74)		13 (28.26)	22 (47.83)	11 (23.91)	
	Urban	43 (27.04)	41 (25.79)	75 (47.17)		47 (29.56)	92 (57.86)	20 (12.58)	
Year of education	No formal education	12 (27.27)	13 (29.55)	19 (43.18)	0.064	16 (36.36)	26 (59.09)	2 (4.55)	0.002^*^
	Class 1-5	33 (23.57)	27 (19.29)	80 (57.14)		38 (27.14)	91 (65.00)	11 (7.86)	
	Class 6-10	42 (33.60)	36 (28.80)	47 (37.60)		37 (29.60)	67 (53.60)	21 (16.80)	
	Class 11-12	10 (23.26)	11 (25.58)	22 (51.16)		18 (41.86)	16 (37.21)	9 (20.93)	
	Graduation	5 (19.23)	4 (15.38)	17 (65.38)		6 (23.08)	12 (46.15)	8 (30.77)	
	Post-Graduation	0 (0.00)	1 (16.67)	5 (83.33)		4 (66.67)	2 (33.33)	0 (0.00)	
Profession	Housewife	18 (42.86)	9 (21.43)	15 (35.71)	0.365	7 (16.67)	33 (78.57)	2 (4.76)	0.001^*^
	Govt. Employee	3 (27.27)	3 (27.27)	5 (45.45)		2 (18.18)	6 (54.55)	3 (27.27)	
	Private Service	24 (25.53)	22 (23.40)	48 (52.06)		35 (37.23)	40 (42.55)	19 (20.21)	
	Business	23 (21.90)	23 (21.90)	59 (56.19)		35 (33.33)	51 (48.57)	19 (18.10)	
	Farmer	14 (29.79)	14 (29.79)	19 (40.43)		19 (40.43)	25 (53.19)	3 (6.3)	
	Retired	8 (24.24)	5 (15.15)	20 (60.61)		8 (24.24)	24 (72.73)	1 (3.03)	
	Unemployment	12 (23.08)	16 (30.77)	24 (46.15)		13 (25.00)	35 (67.31)	4 (7.69)	
Marital status	Married	99 (26.68)	86 (23.18)	186 (50.13)	0.140	116 (31.27)	204 (54.99)	51 (13.75)	0.506
	Single	3 (25.00)	6 (50.00)	3 (25.00)		3 (25.00)	9 (75.00)	0 (0.00)	
	Widowed	0 (0.00)	0 (0.00)	1 (100.00)		0 (0.00)	1 (100.00)	0 (0.00)	
Personal monthly income (BDT)	No	38 (32.76)	31 (26.72)	47 (40.52)	0.117	26 (22.41)	84 (72.41)	6 (5.17)	0.000^*^
	1– ≤ 10,000	19 (26.03)	15 (20.55)	39 (53.42)		19 (26.03)	45 (61.64)	9 (12.33)	
	10,001–20,000	20 (21.51)	21 (22.58)	52 (55.91)		41 (44.09)	41 (44.09)	11 (11.83)	
	20,001–50,000	19 (22.35)	18 (21.18)	48 (56.47)		27 (31.76)	33 (38.82)	25 (29.41)	
	>50,000	6 (35.29)	7 (41.18)	4 (23.53)		6 (5.04)	11 (5.14)	0 (0.00)	
Division	Dhaka	60 (25.10)	55 (23.01)	124 (51.88)	0.825	35 (14.65)	70 (29.29)	134 (56.07)	0.345 (f)
	Chittagong	19 (25.68)	21 (28.38)	34 (45.95)		6 (8.11)	23 (31.08)	45 (60.81)	
	Khulna	6 (40.00)	5 (33.33)	4 (26.67)		2 (13.33)	5 (33.33)	8 (53.33)	
	Rangpur	5 (33.33)	4 (26.67)	6 (40.00)		4 (26.67)	6 (40.00)	5 (33.33)	
	Sylhet	4 (33.33)	1 (8.33)	7 (58.33)		4 (33.33)	3 (25.00)	5 (41.67)	
	Rajshahi	2 (20.00)	3 (30.00)	5 (50.00)		0 (0.00)	5 (50.00)	5 (50.00)	
	Mymensingh	4 (40.00)	1 (10.00)	5 (50.00)		0 (0.00)	3 (30.00)	7 (70.00)	
	Barishal	2 (22.22)	2 (22.22)	5 (55.56)		0 (0.00)	4 (44.44)	5 (55.56)	
Smoking history	Current smoker	22 (18.03)	24 (19.67)	76 (62.30)	0.013^*^	40 (32.79)	64 (52.46)	18 (14.75)	0.691
	Former smoker	40 (28.99)	37 (26.81)	61 (44.20)		46 (33.33)	76 (55.07)	16 (11.59)	
	Never smoker	40 (32.26)	31 (25.00)	52 (42.74)		33 (26.61)	74 (59.68)	17 (13.28)	
Alcohol consumption	No	100 (26.95)	89 (23.72)	185 (49.33)	0.841	117 (31.28)	208 (55.61)	49 (13.10)	0.663
	yes	2 (20.00)	3 (30.00)	5 (50.00)		2 (20.00)	6 (60.00)	2 (20.00)	
Drug abuse	No	101 (26.79)	91 (24.14)	185 (49.07)	0.502	115 (30.50)	213 (56.50)	49 (13.00)	0.081
	yes	1 (14.29)	1 (14.29)	5 (71.43)		4 (57.14)	1 (14.29)	2 (28.57)	
Chronic disease	DM	13 (25.00)	12 (23.08)	27 (51.92)	0.050^*^	22 (42.31)	23 (44.23)	7 (13.73)	0.115
	Hypertension	17 (25.00)	21 (30.88)	30 (44.12)		21 (30.88)	39 (57.35)	8 (11.76)	
	Both HTN and DM	15 (39.47)	13 (34.21)	10 (26.32)		6 (15.79)	29 (76.32)	3 (7.89)	
	None	57 (25.22)	46 (20.35)	123 (54.42)		119 (30.99)	214 (55.73)	33 (14.60)	
STEMI	No	60 (29.27)	60 (29.21)	*85 (41.46)*	0.001^*^	59 (28.79)	121 (59.02)	25 (12.20)	0.379
	Yes	42 (23.46)	32 (17.88)	*105 (58.66)*		60 (33.52)	93 (51.96)	26 (14.53)	
NSTEMI	No	90 (28.30)	82 (25.79)	146 (45.91)	0.003^*^	119 (30.99)	180 (56.60)	41 (12.89)	0.739
	Yes	12 (18.18)	10 (15.15)	44 (66.67)		22 (33.33)	34 (51.52)	10 (15.15)	
Old MI	No	81 (25.88)	70 (22.36)	162 (51.76)	0.144	96 (30.67)	174 (55.59)	43 (13.74)	0.849
	Yes	21 (29.58)	22 (30.99)	28 (39.44)		23 (32.39)	40 (56.34)	8 (11.27)	
Recent MI	No	99 (26.83)	89 (24.12)	181 (49.05)	0.703	112 (30.35)	209 (56.64)	48 (13.01)	0.154
	Yes	3 (20.00)	3 (20.00)	9 (60.00)		7 (46.67)	5 (33.33)	3 (20.00)	
Systemic hypertension	No	73 (25.61)	65 (22.81)	147 (51.58)	0.373	94 (32.98)	156 (54.74)	35 (12.28)	0.294
	Yes	29 (29.29)	27 (27.27)	43 (43.43)		25 (25.25)	58 (58.59)	16 (16.16)	
Diabetes mellitus	No	80 (26.67)	66 (22.00)	154 (51.33)	0.207	93 (31.00)	166 (55.33)	41 (13.67)	0.908
	Yes	22 (26.19)	26 (30.95)	36 (42.86)		26 (30.95)	48 (57.14)	10 (11.90)	
ALVF	No	82 (25.23)	83 (25.54)	160 (49.23)	0.162	100 (30.77)	178 (54.77)	47 (14.46)	0.290
	Yes	20 (33.90)	9 (15.25)	30 (50.85)		19 (32.20)	36 (61.02)	4 (6.78)	
Unstable angina	No	86 (27.74)	68 (21.94)	156 (50.32)	0.148	103 (33.23)	168 (54.19)	39 (12.58)	0.144
	Yes	16 (21.62)	24 (32.43)	34 (45.95)		16 (21.62)	46 (62.16)	12 (16.22)	
Stable angina	No	99 (26.54)	90 (24.13)	184 (49.33)	0.896	116 (31.10)	209 (56.03)	48 (12.87)	0.379
	Yes	3 (27.27)	2 (18.18)	6 (54.55)		3 (27.27)	5 (45.45)	3 (27.27)	
Heart valve disease	No	91 (25.42)	85 (23.74)	182 (50.84)	0.096	109 (30.45)	198 (55.31)	51 (14.25)	0.077
	Yes	11 (42.31)	7 (26.92)	8 (30.77)		10 (38.46)	16 (61.54)	0 (0.00)	

*MI, Myocardial infarction; STEMI, ST-elevation myocardial infarction; NSTEMI, Non-ST-Elevation Myocardial Infarction; ALVF, Acute Left Ventricular Failure; BMI, Body mass index; OR, Odds Ratio; CI, Confidence Interval; BDT, Bangladesh taka*.

* *Significant outcomes were indicated by bold values*.

### Factors Associated With Depression and Anxiety

We found an association between anxiety and BMI (*p* = 0.008), area of residence (*p* = 0.031), an education level (*p* = 0.049) and smoking history (*p* = 0.013). People in the group with an income of more than >50000 bdt were found to have less anxiety than those in the other groups. However, these findings were only significant among patients who had an ECG finding indicating STEMI (*p* = 0.001) and NSTEMI cardiac diagnosis (*p* = 0.003) (Table 4).

However, there was an association between depression with age (*p* = 0.000), years of education (*p* = 0.002), occupation (*p* = 0.001) and individual monthly income (*p* = 0.000) on bi-variate analysis Post-graduation group had the highest level (83.33%) of abnormal level of anxiety. In terms of education, persons who had completed their post-graduation without depression also had the highest rate of depression (67%), and aberrant depression was more common in the graduating group (31%) (Table 4). Those variables with *p*-values <0.05 were incorporated into the multivariable model.

For multivariable analysis, both anxiety and depression were categorized into two groups: one normal group and the other abnormal group (combination of both borderline abnormal and abnormal) (Table 4). Multivariable logistic regression found residence, age, profession, and income to be statistically significantly associated with anxiety after controlling for the other factors found to be significant at the univariate level. In the case of depression, multivariable logistic regression revealed only gender as being significantly associated after controlling for confounders (Table 5).

**Table 5:** Factors associated with anxiety and depression in multivariable logistic regression.

**Variables**	**Category**	**Anxiety**				**Depression**			
		**Crude OR (95%CI)**	***p*-value**	**Adjusted OR (95%CI)**	***p***	**Crude OR (95%CI)**	***p*-value**	**Adjusted OR (95%CI)**	***p***
Gender	Male	0.52 (0.27–0.99)	0.050	1.46 (0.09–23.9)	0.788			1.46e−06 (0)	0.006^*^
	Female	Ref	Ref	Ref		Ref	Ref	ref	
Age	Up to 40	0.74 (0.38–1.43)	0.380	0.65 (0.29–1.46)	0.300	0.38 (0.1–1.11)	0.079	0.60 (0.18–1.99)	0.410
	41–50	0.56 (0.31–0.99)	0.048	0.46 (0.23–0.93)	0.033^*^	0.23 (0.90–0.60)	0.003	0.39 (0.14–1.09)	0.075
	51–60	0.61 (0.35–1.06)	0.085	0.52 (0.27–1.02)	0.057	0.84 (0.29–2.39)	0.743	1.21 (0.39–3.78)	0.737
	61 to above	Ref	Ref	Ref		Ref	Ref	Ref	Ref
Residence	Rural	1.05 (0.68–1.62)	0.802	1.11 (0.66–1.86)	0.691				
	Semi-urban	0.35 (0.17–0.71)	0.004	0.42 (0.19–0.93)	0.034^*^				
	Urban	Ref	Ref	Ref					
Year of education	No formal education					Ref	Ref	Ref	
	Class 1–5					0.56 (0.11–2.62)	0.460	0.59 (0.12–2.97)	0.515
	Class 6–10					0.24 (0.05–1.05)	0.058	0.27 (0.05–1.33)	0.116
	Class 11–12					0.18 (0.04–0.89)	0.035	0.32 (0.05–1.85)	0.204
	Graduation					0.11 (0.20–0.56)	0.008	0.21 (0.03–1.30)	0.094
	Post-graduation					1	-	1	-
Profession	Housewife	2.30 (0.76–2.91)	0.027	0.42 (0.02–9.85)	0.593	4.42 (0.98–19.89)	0.053	2.90e (0)	0.982
	Govt. employee	1.53 (0.37–1.85)	0.498	2.35 (0.57–9.56)	0.232	0.59 (0.14–2.43)	0.464	0.54 (0.10–2.83)	0.474
	Private service	1.22 (0.44–5.36)	0.469	1.41 (0.74–2.68)	0.294	0.87 (0.43–1.77)	0.705	0.86 (0.38–1.92)	0.719
	Business	Ref	Ref	Ref	Ref	Ref	Ref	Ref	Ref
	Farmer	1.89 (1.10–4.83)	0.074	1.88 (0.82–4.32)	0.132	3.24 (0.91–11.54)	0.070	1.85 (0.45–7.51)	0.389
	Retired	0.83 (0.37–1.85)	0.655	0.42 (0.02–9.85)	0.009^*^	7.06 (0.91–54.99)	0.062	2.16 (0.17–26.24)	0.544
	Unemployment	1.49 (0.79–2.92)	0.237	0.20 (0.04–1.00)	0.051	2.65 (0.85–8.24)	0.092	0.60 (0.05–6.63)	0.679
Personal monthly income (BDT)	No	Ref	Ref	ref		Ref	Ref	ref	
	1– ≤ 10,000	0.59 (0.32–1.07)	0.084	0.16 (0.04–0.67)	0.012^*^	0.38 (0.13–1.14)	0.085	0.34 (0.04–2.64)	0.307
	10,001–20,000	0.54 (0.30–0.93)	0.027	0.11 (0.02–0.55)	0.006^*^	0.41 (0.14–1.14)	0.000	0.604 (0.06–5.87)	0.684
	20,001–50,000	0.52 (0.30–0.93)	0.027	0.11 (0.02–0.53)	0.006^*^	0.13 (0.05–0.34)	0.000	0.21 (0.02–2.00)	0.178
	>50,000	2.21 (0.68–7.20)	0.187	0.40 (0.05–2.77)	0.355	1	–	1	-
Smoking history	Current smoker	0.45 (0.27–0.75)	0.002	0.60 (0.32–1.14)	0.123				
	Former smoker	0.94 (0.57–1.54)	0.812	1.11 (0.60–2.06)	0.727				
	Never smoker	Ref	Ref	ref	Ref				
Chronic disease	DM	1.10 (0.60–2.02)	0.744	1.44 (0.71–2.94)	0.307				
	Hypertension	1.51 (0.87–2.61)	0.137	1.54 (0.83–2.87)	0.170				
	Both HTN and DM	3.34 (1.55–7.20)	0.002	2.85 (1.22–6.65)	0.015^*^				
	None	Ref	Ref	Ref	Ref				
STEMI	No	Ref	Ref	Ref	Ref				
	Yes	0.49 (0.33–0.75)	0.001	0.59 (0.40–1.18)	0.180				
NSTEMI	No	Ref	Ref	Ref	Ref				
	*Yes*	0.42 (0.24–0.74)	0.003	0.66 (0.33–1.34)	0.259				

*MI, Myocardial infarction; STEMI, ST-elevation myocardial infarction; NSTEMI, Non-ST-Elevation Myocardial Infarction; ALVF, Acute Left Ventricular Failure; OR, Odds Ratio; CI, Confidence Interval; BDT, Bangladesh taka*.

* *Significant results*.

## Discussion

The prevalence of depression and anxiety level was moderately high in this study. An abnormal degree of anxiety affected 49.5%, whereas borderline anxiety affected 23.9% of the study population. This conclusion resembles that of the Brazilian population (30), where it was found that 48.4% of CAD patients were anxious. Anxiety levels among CAD patients were slightly more pronounced in our study, which might be related to unemployment following sickness, level of illiteracy, a lack of knowledge about the prognosis of CAD, or even lack of counseling resources in a developing country context. In our study, around 55.7% of patients had borderline depression, and 13.2% had abnormal depression, whereas studies in Brazil (30) and Germany (31) revealed that 26.4 and 5.9% of CAD patients, respectively, had depression. Depression was found to be much higher among CAD patients in our study which could be due to a lack of information and limited access to quality health care, including the huge out of pocket expenditure.

The sex of patients was also shown to be substantially related to their degree of anxiety with males having a higher level of anxiety than females. Similar findings were seen in Brazil (32), but a study from America (33) found that female CAD patients had more significant anxiety than males. Furthermore, our study linked family income and occupation status to CAD patients' anxiety levels, with patients whose yearly family income was insufficient funds experiencing higher anxiety levels. However, research from Pakistan (34) found no link between anxiety and CAD patients' socioeconomic levels. The disparity in results might be attributed to differences in sample size of the study population.

Our study also indicated that anxiety levels were related to age, with 23% lower anxiety levels in the younger (41–50) group than in the 61 and above age group. Our findings are consistent with research done in the United States (33), which also found that age was strongly related to anxiety levels. Patients with comorbid conditions such as HTN and DM were at 2.8 times higher risk than patients having no comorbidity. Our findings are consistent with research done in India that found a strong link between CAD patients' anxiety and their comorbid illness. We did not find any predictors strongly predicting depression after controlling for confounders in our study.

There is limited information about anxiety and depression in hospitalized cardiology departments particularly during the COVID-19 era which itself could add to the anxiety and depression experienced by patients and limited to no prospective evidence about heart disease patient mental well-being in Bangladesh. Our study provides essential information on undiagnosed anxiety and depression among heart disease patients admitted to a Bangladeshi hospital. The study also suggests the potential for further research studies to evaluate the clinical significance of anxiety and depression on individuals with cardiovascular disease, including prognosis and management of CVD.

## Strength and Limitation

It is the first study in Bangladesh to assess the mental health status of hospitalized heart disease patients. However, the cross-sectional form of the study makes it very difficult to establish valid inferences. A long-term follow-up should be conducted to see if the study's findings are consistent in similar settings and have any bearing on the management and prognosis of heart disease. Including an interaction term in multivariable regression would be more informative, but due to the limited sample size, this could not be done. In addition, and our main goal was to assess the prevalence and predictors of anxiety and depression among hospitalized heart disease patients which has been presented taking into consideration major confounders.

Despite its widespread use, the HADS has significant drawbacks. For starters, a cutoff score of 11 on the HADS depression subscale (HADS-D) has been found to have a sensitivity of just 38% for diagnosing clinical depression, implying that most of the depression in the study's population went undiscovered (35). In addition, unlike other self-report questionnaires such as the Patient Health Questionnaire (PHQ), the HADS does not capture somatic symptoms, including fatigue and sleeplessness. Over 30 years ago, the idea was that physical instead of mental disorders could cause fatigue and insomnia (35).

## Conclusion

Our findings indicate that health care providers, particularly cardiologists and nurses, should take extra care to detect and evaluate all heart disease patients for level of anxiety and depression in a clinical setting. There is a need to develop a quick screening approach in hospitals dealing with cardiovascular inpatients to identify those needing extra evaluation and care.

## Data Availability Statement

The original contributions presented in the study are included in the article/supplementary material, further inquiries can be directed to the corresponding author.

## Ethics Statement

The studies involving human participants were reviewed and approved by North-South University's Institutional Review Board/Ethical Review Committee also provided ethical approval for this project (2021-IRB-0502). The patients/participants provided their written informed consent to participate in this study.

## Author Contributions

Conceptualization: MAm, MAh, and NK. Data curation: MAm. Formal analysis and methodology: MAm and SN. Project administration and visualization: MAh and NK. Writing—original draft: MAm and NK. Writing—review and editing: MAm, SN, and NK. All authors approved the submitted version for publication.

## Conflict of Interest

The authors declare that the research was conducted in the absence of any commercial or financial relationships that could be construed as a potential conflict of interest.

## Publisher's Note

All claims expressed in this article are solely those of the authors and do not necessarily represent those of their affiliated organizations, or those of the publisher, the editors and the reviewers. Any product that may be evaluated in this article, or claim that may be made by its manufacturer, is not guaranteed or endorsed by the publisher.
